# Individualized Management of Traumatic Limb Pseudoaneurysms: A Case-Based Comparison of Endovascular and Open Repair

**DOI:** 10.7759/cureus.89065

**Published:** 2025-07-30

**Authors:** Bistra Boneva, Boris Ilchev

**Affiliations:** 1 Vascular Surgery, National Cardiology Hospital, Sofia, BGR; 2 Vascular Surgery, Acıbadem City Clinic Tokuda Hospital, Sofia, BGR

**Keywords:** endovascular repair, individualized treatment strategy, limb ischemia, open surgical repair, traumatic pseudoaneurysm, vascular trauma

## Abstract

Traumatic limb pseudoaneurysms are rare vascular complications that can cause acute limb ischemia and require urgent treatment. Traditionally addressed through open surgical repair, advances in endovascular techniques have introduced less invasive options, particularly beneficial for high-risk patients. This article presents two cases illustrating individualized decision-making in choosing between endovascular and open surgical repair.

A comparative case-based approach was employed. The first case involved a 94-year-old male with multiple comorbidities and a femoral pseudoaneurysm following blunt trauma. Due to anesthetic risk, endovascular repair with overlapping stent grafts was performed. The second case described a 34-year-old male with a delayed presentation of a traumatic axillary artery pseudoaneurysm. Open surgical exploration and autologous vein graft interposition were undertaken.

In the elderly patient, the endovascular approach achieved rapid exclusion of the pseudoaneurysm, with restoration of limb perfusion and no postoperative bleeding. In the younger patient, open surgery successfully re-established arterial continuity and function. Some residual sensory deficits persisted due to delayed presentation, but follow-up showed good perfusion and no signs of ischemia.

These cases highlight the importance of patient-specific management. Endovascular repair offers a minimally invasive solution with lower perioperative risk, especially suitable for elderly or frail patients. Open surgery remains essential for durable reconstruction in young, otherwise healthy individuals or in anatomically complex cases. Selection of the optimal approach depends on age, comorbidities, pseudoaneurysm location, and time from injury to presentation.

Traumatic pseudoaneurysms require individualized management strategies. Minimally invasive repair is generally reserved for patients at elevated surgical risk, whereas open repair remains a durable option for younger, healthier individuals. These cases reflect the need for flexible, evidence-informed decision-making in vascular trauma. With limited large-scale data and standardized protocols, further research is needed to guide optimal treatment strategies.

## Introduction

Limb pseudoaneurysms are uncommon but clinically important and significant vascular complications that can arise from trauma, surgical interventions, or spontaneous occurrences. Though rare, they carry the risk of significant morbidity if not promptly diagnosed and treated. They involve a defect in the arterial wall, allowing blood to escape and form a contained, pulsatile hematoma that communicates with the arterial lumen. The clinical presentation can vary from asymptomatic and incidental findings to severe limb ischemia, necessitating prompt diagnosis and intervention [1].

Traditionally, open surgical repair has been the mainstay treatment for limb pseudoaneurysms, offering direct visualization and repair of the arterial defect [2].

However, advancements in endovascular techniques, such as the placement of covered stents, have emerged as less invasive alternatives that can be particularly beneficial in high-risk surgical candidates. Endovascular repair, involving the placement of covered stents, has shown promising results in terms of technical success and reduced perioperative morbidity [3]. In addition to comorbidities and overall surgical risk, the choice between open and endovascular repair in trauma patients may also be influenced by concomitant injuries, such as associated venous trauma or long bone fractures, which can dictate surgical accessibility and urgency.

We present two distinct cases, each successfully managed using either an endovascular or an open surgical approach. These cases underscore the critical importance of proficiency in both techniques and highlight the necessity of a case-specific strategy in addressing these rare but potentially life-threatening situations.

## Case presentation

**Figure 1:**
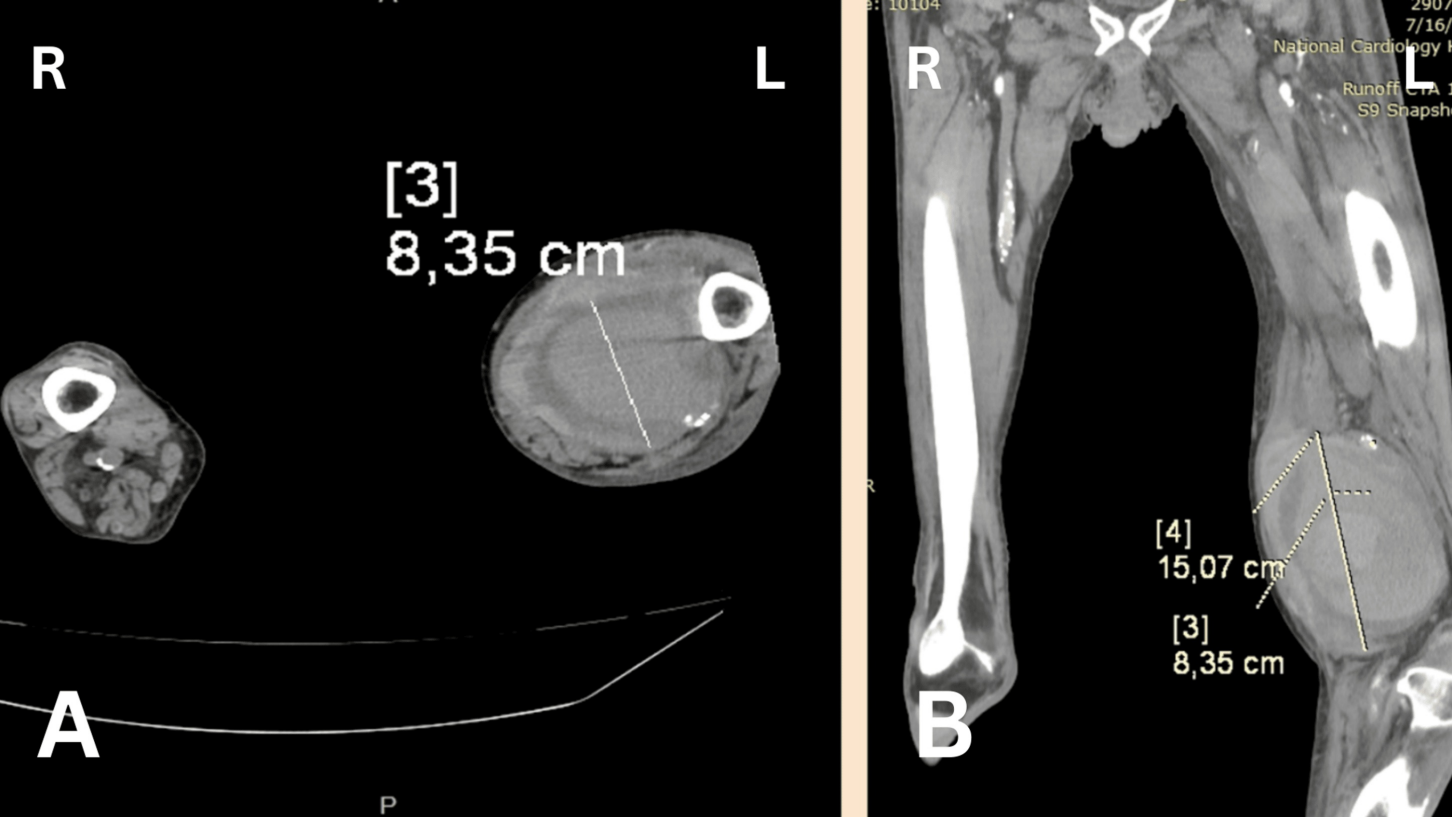
CT angiography of the popliteal artery pseudoaneurysm. A: Transversal view. B: Sagittal view.

A 94-year-old male with а history of arterial hypertension, implantation of a pacemaker, and achalasia of the esophagus presented to the emergency department. The patient had sustained a blunt trauma of the left femur 10 days ago with the formation of a pulsatile mass, which had progressively enlarged over the days, resulting in the inability to stand on his feet at the time of examination. On arrival at the emergency department, the patient presented with symptoms of pallor and numbness of the left foot. Upon examination, there were clinical signs of acute ischemia of the left lower extremity with cyanosis of the distal phalanges I-V and lack of pulse of the popliteal artery distally. Neurological examination was limited, but numbness of the foot was noted, raising concern for early ischemic neuropathy. A large pulsating formation at the distal portion of the left femur was observed. An ultrasound examination verified a pulsating hematoma. Computed tomography angiography (CTA) was performed, and verified an aneurysm with a maximum transverse diameter of 84 mm and longitudinal size of 15 mm (Figure 1).

**Figure 2:**
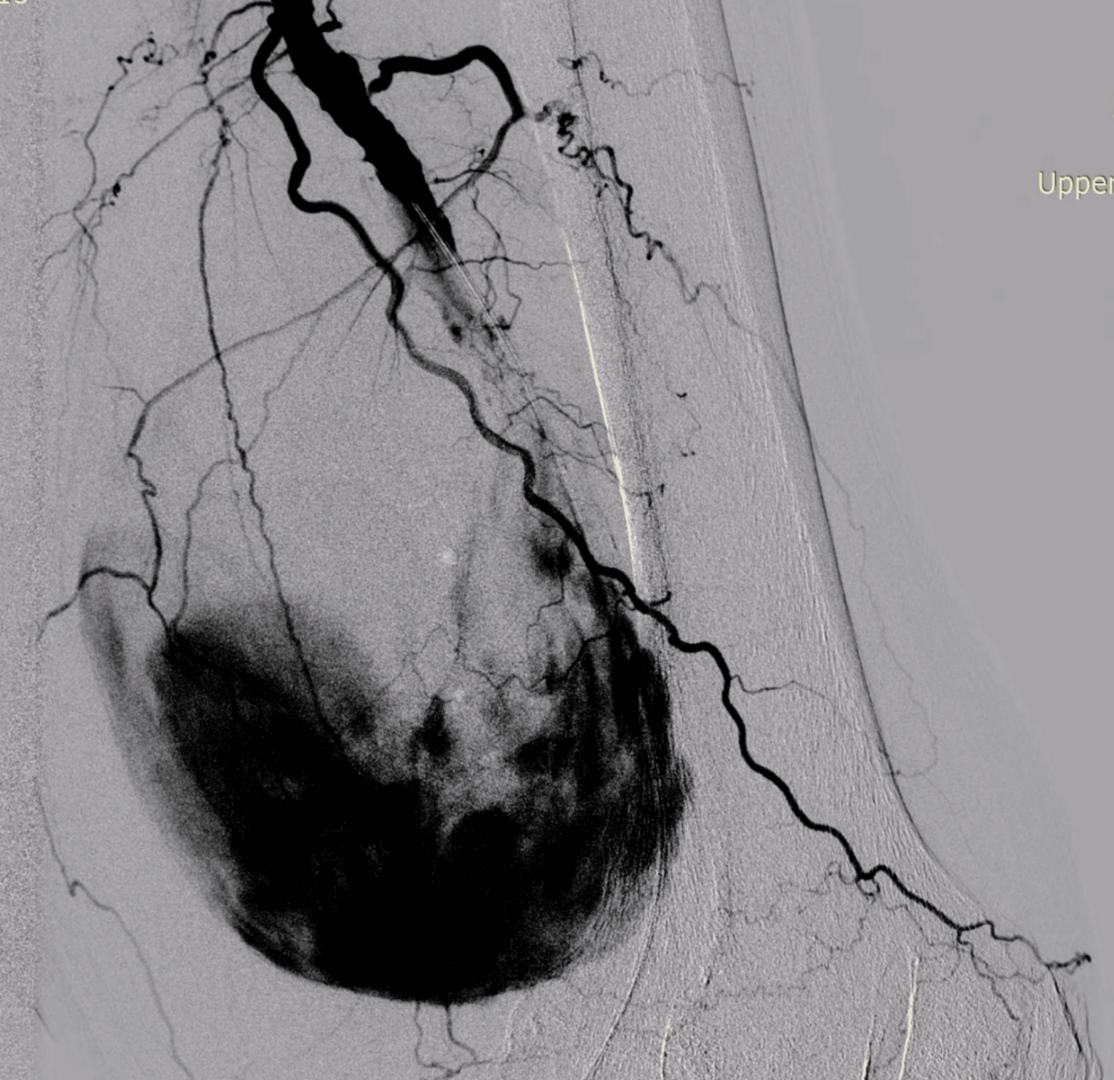
Selective angiography verified a pseudoaneurysm located medial to the artery, with the blood flow entry of the P2 segment of the popliteal artery.

The patient was considered at very high risk for any type of anesthesia other than local, which led to the decision for an endovascular approach. This decision was based on his advanced age, history of arterial hypertension, permanent pacemaker implantation, and overall frailty, which collectively posed significant cardiovascular risk under general anesthesia. Due to the high anesthetic risk and advanced age, combined decompression and hematoma evacuation were deferred in favor of a staged approach. An antegrade access of the left common femoral artery (CFA) was acquired. Selective angiography verified a pseudoaneurysm located medial to the artery with the blood flow entry of the P2 segment of the popliteal artery (PA) (Figure 2).

**Figure 3:**
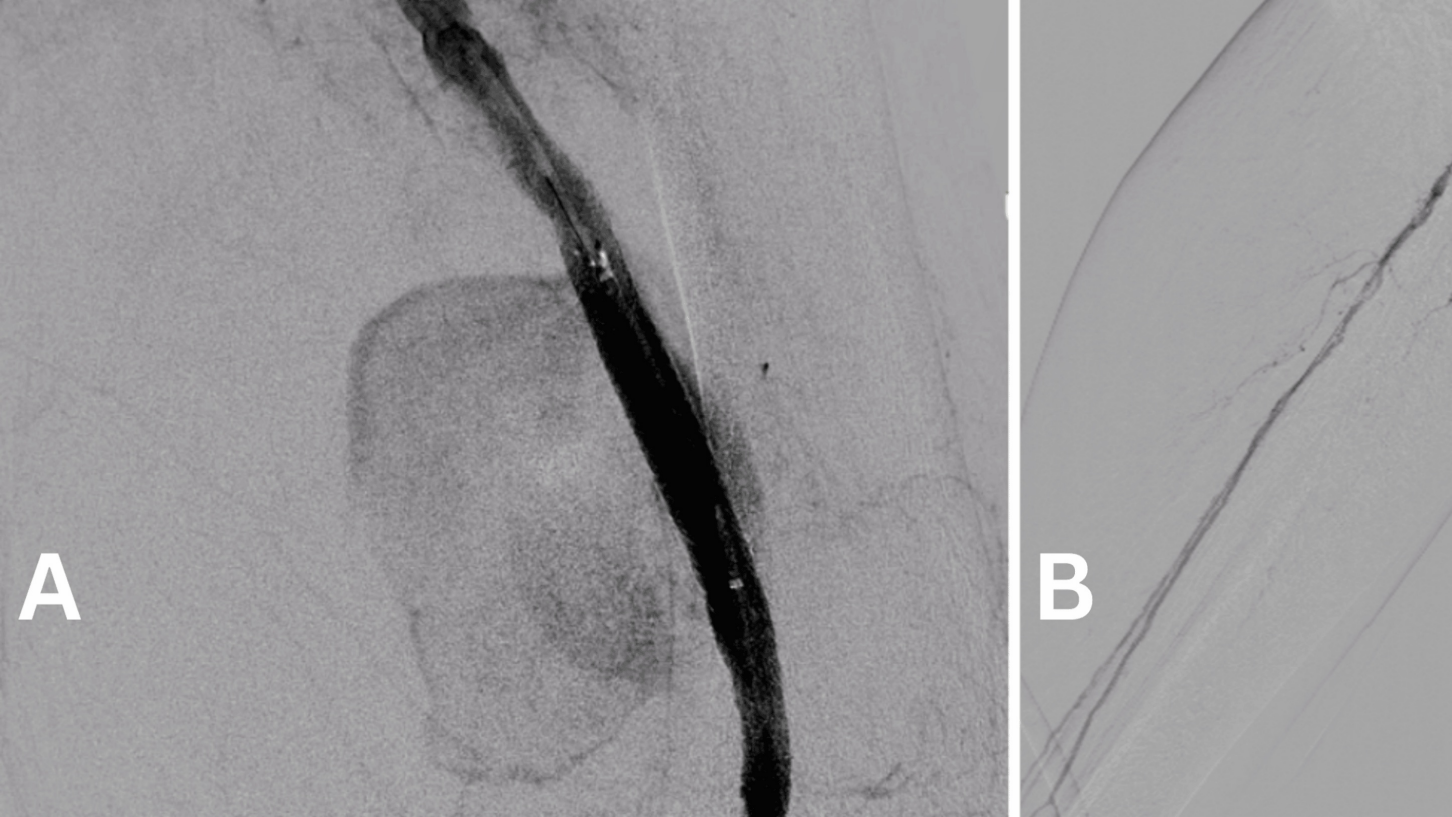
Almost complete isolation of the pseudoaneurysm with minimal endoleak at the site of overlapping of the two stent grafts and patent single vessel tibial outflow. A: Almost complete isolation of the pseudoaneurysm with minimal endoleak at the site of overlapping of the two stent grafts. B: Single tibial artery outflow to the foot.

A guidewire was placed in the distal popliteal, and two stent grafts were implanted - Begraft 8/57 mm (Bentley InnoMed, Hechingen, Germany) and Advanta 8/38 mm (Getinge, Gothenburg, Sweden) (the only off-the-shelf devices available at our center at that time). The control angiography showed almost complete isolation of the pseudoaneurysm with minimal endoleak at the site of overlapping of the two stent grafts. P3 segment of the popliteal artery was visualized with partially reduced lumen distally and gracile tibial arteries with a patent posterior tibial artery all the way to the ankle (Figure 3).

The next day, the patient underwent a new ultrasound examination, which showed no active bleeding. At this point, hematoma evacuation was performed under local anesthesia, with no clinical signs of compartment syndrome and improved distal perfusion. The patient was discharged with a pulsatile popliteal artery, and the foot was compensated. The initial hemorrhagic shock state of the patient during the perioperative period was managed successfully. Postoperatively, the patient was started on dual antiplatelet therapy (aspirin and clopidogrel) for one month, followed by clopidogrel monotherapy, tailored to optimize stent patency while minimizing bleeding risk.

Open surgical management

**Figure 4:**
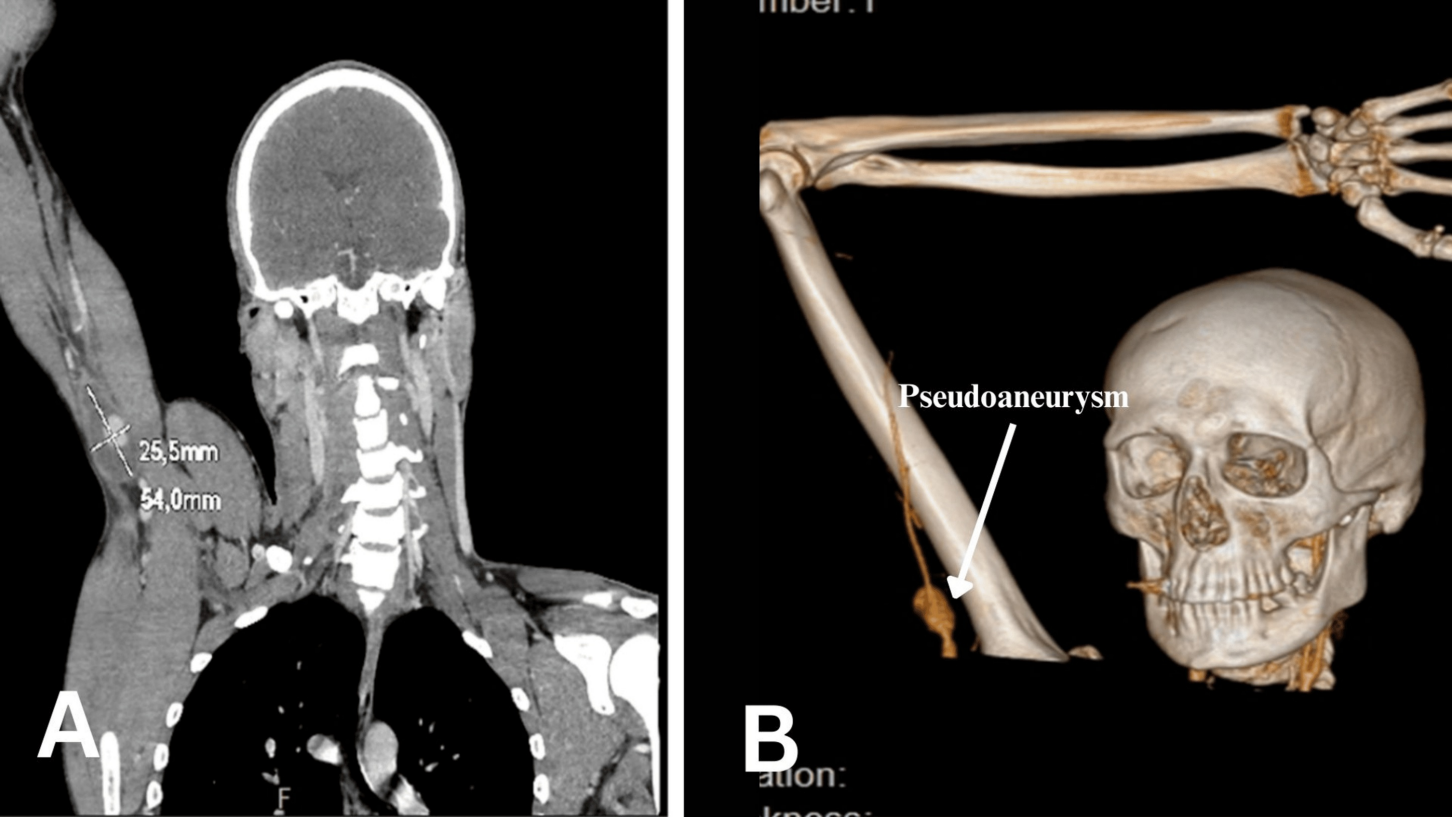
Pseudoaneurysm of the right axillary artery. A: Coronal plane. B: 3D reconstruction.

A 34-year-old male without any comorbidities presented to the emergency department. The patient had sustained a blunt trauma to the right humeral joint two months ago. He reported a sensation of a pulsatile formation in the axillary fossa after the accident, which he had neglected at the time. This delay in seeking care may reflect a lack of early alarming symptoms and a tendency, especially in younger patients, to underestimate the severity of vascular injury. On arrival at the emergency department, the patient presented with symptoms of pallor, numbness, and pain in the right forearm, palm, and fingers. On examination, there were clinical signs of acute ischemia of the right upper extremity with cyanosis of the distal phalanges of II-III-IV fingers and lack of pulse of the brachial, radial, and ulnar arteries. A large pulsating formation in the axillary fossa was observed. An ultrasound examination verified a pseudoaneurysm of the axillary artery with a size of 25/29 mm, a short occlusion of the transition to the brachial artery, and at the bifurcation of the brachial artery. A CTA was performed and verified a pseudoaneurysm of the right axillary artery with a size of 54/26/42 mm and an occlusion of the right brachial artery (Figure 4).

**Figure 5:**
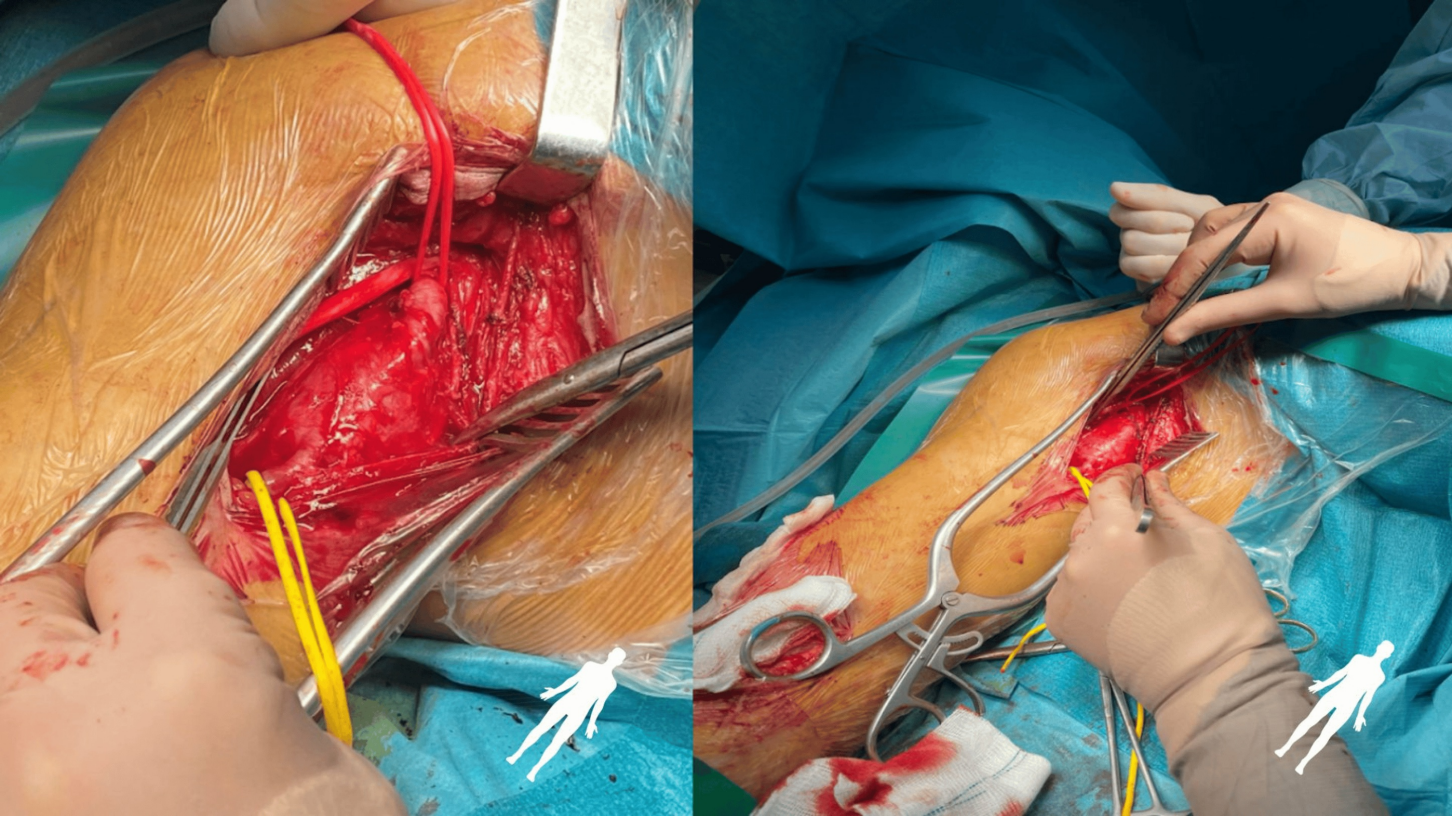
Surgical exposure and dissection of the axillary artery proximal to the pseudoaneurysm at the axillary fossa.

The patient was managed with an open surgical approach and emergent exploration. The bifurcation of the brachial artery was dissected at the antecubital fossa. The brachial artery proximally and the radial and ulnar arteries distally were controlled with double-looped vascular slings. After systemic heparinization, a transverse arteriotomy was performed. A 4 fr Fogarty catheter was introduced proximally, and embolectomy was performed, verifying mixed thrombi with intimal fragments originating from the pseudoaneurysmal sac. Excellent in-flow was achieved afterwards. The radial artery was impenetrable with an embolectomy catheter. Empiric embolectomy of the ulnar artery with a 3 fr Fogarty at 25 cm distal to the origin was performed with no thrombus evacuation and satisfactory backflow. The arteries were flushed with heparinized saline. Surgical exposure and dissection of the axillary artery proximal to the pseudoaneurysm at the axillary fossa was performed, as well as brachial artery exposure at the transition with the axillary artery just distal to the pseudoaneurysm (Figure 5).

**Figure 6:**
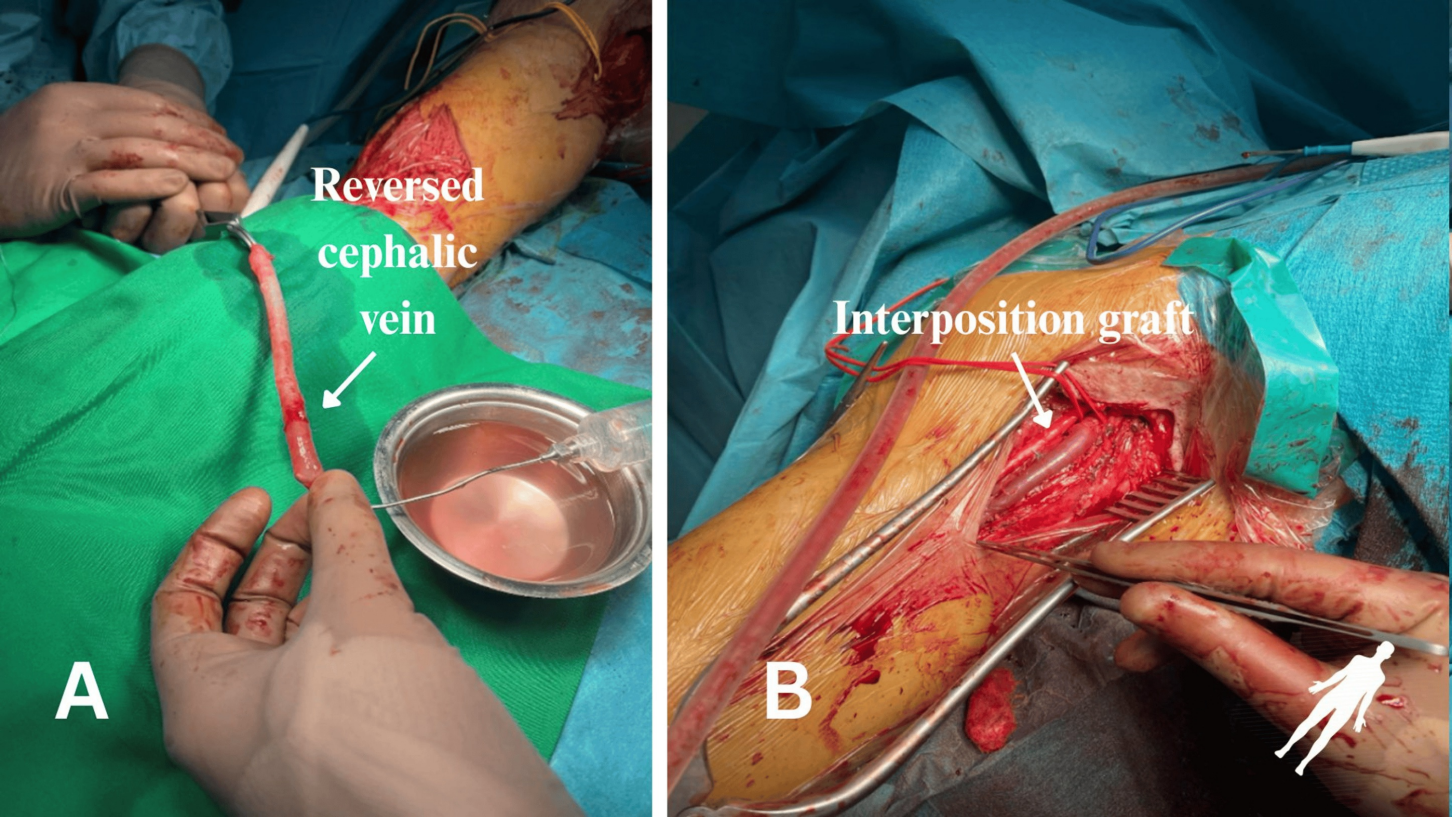
Preparation and construction of the interposition graft. A: Preparation of the cephalic vein, which is reverted to construct the interposition bypass. B: Final result showcasing the interposition graft.

A decision for an autovenous graft interposition was made. With an additional incision at the middle portion of the brachium, the cephalic vein was harvested and reversed. The pseudoaneurysm was ligated and excluded proximally and distally. Interposition of the autovenous graft was performed with two end-to-end anastomoses. At the completion of the graft, limb perfusion was restored with a pulse of the brachial artery (Figure 6).

Ultrasound blood flow of the radial and ulnar arteries at the wrist and the palmar arch was evaluated postoperatively, recording 100 mmHg blood pressure of the ulnar artery and 0 mmHg of the radial artery, with a systemic blood pressure of 120 mmHg. However, due to prolonged ischemic time prior to presentation, partial numbness of the distal phalanges of II-III-IV fingers persisted. The patient was discharged on oral anticoagulation with acenocoumarol, with plans for re-evaluation at three months and possible transition to antiplatelet therapy thereafter. Duplex ultrasonography surveillance was scheduled at one, three, six, and 12 months postoperatively. At one-month follow-up, the patient was examined with no symptoms or clinical signs of ischemia. Ultrasound examination recorded 90 mmHg blood pressure of both radial and ulnar arteries at 120 mmHg systemic blood pressure.

## Discussion

Pseudoaneurysms (PAs) are localized defects of the vessel wall that result in turbulent blood flow and hematoma formation [4]. In contrast to true aneurysms, PAs involve only one or two of the normal three arterial wall layers, most often confined by the adventitia alone [5]. Disruption of the arterial wall leading to PA formation can arise from trauma, inflammation, infection, or iatrogenic injury [4,5]. Traumatic PAs may follow either blunt or penetrating arterial injury. Although many remain asymptomatic, they can present with swelling, pain, or a mass effect [6]. Because the arterial wall is compromised, PAs carry a high risk of enlargement and rupture, potentially causing life-threatening hemorrhage and death, and thus require prompt intervention. Historically, treatment was limited to surgical techniques, such as arterial reconstruction, ligation, and resection of the affected tissue [4,7,8]. Given the unstable condition of these patients, morbidity and mortality rates for surgical repair have reached as high as 50% [9,10].

Traumatic pseudoaneurysms of the limbs pose a unique challenge that necessitates an individualized treatment strategy. To date, the literature on this pathology remains limited, primarily comprising isolated case reports. Consequently, the decision to pursue an endovascular or open surgical approach is largely driven by patient-specific factors, including anatomical considerations, aneurysm location, and clinical expertise [11]. Other key factors include comorbidities, anesthesia risk, concomitant injuries, and the presence of complications such as neurological deficits or compartment syndrome.

Management of pseudoaneurysms, particularly in the context of acute limb ischemia, imperatively requires some kind of repair. The American College of Cardiology and the American Heart Association recommend the repair of symptomatic femoral artery aneurysms to mitigate the risks of thromboembolic complications and limb loss [12]. In this case, an endovascular approach was chosen, supported by recent literature indicating that endovascular repair can be effective and associated with fewer complications compared to open surgery [13,14].

In our experience, the endovascular technique, characterized by stent graft placement, appeared advantageous for the high-risk, elderly patient by reducing perioperative stress and offering a minimally invasive option for rapid pseudoaneurysm exclusion. Nonetheless, the minor endoleak observed post procedure highlights the potential need for vigilant postoperative surveillance and, in some cases, adjunctive interventions. This approach aligns with findings from studies demonstrating that endovascular stenting provides immediate symptom relief while reducing perioperative complications [15].

In recent literature, hybrid endovascular-to-open techniques have been increasingly utilized in anatomically challenging or high-risk vascular injuries. For example, Daskalov and Ilchev described a 62-year-old patient with a traumatic common carotid artery pseudoaneurysm who was successfully treated in a single operative session: an initial covered stent deployment provided hemorrhage control and maintained perfusion, permitting safe subsequent open resection and autologous vein graft reconstruction [16]. This case underscores the versatility and strategic advantage of combining modalities, the very principle applied in our femoral and axillary pseudoaneurysm cases.

Recent advances in imaging and endovascular techniques have enhanced the detection and treatment of PAs in trauma patients [4]. However, there remains a paucity of data specifically examining clinical outcomes following endovascular intervention for traumatic pseudoaneurysms. Previous studies encompassing PAs of diverse origins (e.g., infection, iatrogenic injury, trauma, and inflammation) report clinical success rates of 85.7%-100% and technical success rates of 71.4%-100% [17-20].

Head-to-head comparisons of endovascular and open surgical interventions have demonstrated that endovascular approaches are associated with shorter procedure times, reduced intraoperative blood loss, fewer transfusion requirements, and shorter hospital stays for both true aneurysms and pseudoaneurysms [7,8]. These studies also observed no significant differences between the two modalities in terms of technical or clinical success, major complication rates, or overall survival [7,8]. While endovascular repair is advantageous in selected cases, particularly in elderly or comorbid patients, its long-term durability compared to open surgery remains under ongoing investigation. Some reports suggest a higher risk of re-thrombosis and secondary interventions in certain anatomic settings. In contrast, open surgical repair, especially with autologous vein grafts, is generally favored for durability, particularly in younger, lower-risk patients. Conversion to open surgery during endovascular procedures is uncommon but can be required in the event of technical failure, extensive thrombus burden, or inadequate lesion exclusion. Therefore, while endovascular management is an excellent option for selected cases, open surgical repair remains the preferred standard in scenarios demanding long-term durability. Furthermore, other analyses have underscored the lower risk profile of endovascular management compared to traditional surgery, along with the ability to precisely localize the pseudoaneurysm and assess collateral circulation [5,21,22].

In our opinion, the endovascular approach for managing the pseudoaneurysm in this elderly patient with multiple comorbidities was appropriate and aligns with current evidence from recent studies. In our particular case, the endovascular approach yielded good results as it was effective in managing the hemorrhagic shock and saved the patient from critical limb ischemia [13-15]. However, the single-limb nature of the repair and short follow-up period must be noted when interpreting the outcome.

Traumatic injuries to the axillary artery constitute only 2.9-9% of all major arterial traumas [23]. This condition often manifests late rather than immediately post injury [24], as illustrated by our case, which developed an axillary artery pseudoaneurysm two months after the initial trauma. The absence of overt early signs of arterial damage can lower clinical vigilance, so maintaining a high index of suspicion is essential to avert complications like brachial plexus compression [24]. As noted by other authors, pseudoaneurysms may present either immediately or in a delayed fashion, due to initially small lesion size and the presence of robust collateral circulation in the upper extremity [25]. In our case, we can speculate, mainly based on his anamnesis alone, that the patient developed an initially small aneurysm, which he initially neglected.

The precise mechanism by which axillary artery pseudoaneurysms develop remains uncertain, though the vessel’s anatomical relationships are thought to increase its vulnerability. It may be predisposed to injury by being tethered between the anterior and posterior circumflex and scapular arteries. Alternatively, hyperabduction of the humeral head may force the artery against the pectoralis minor, and individual variations in its course could further heighten the risk of trauma [26].

Managing axillary artery injuries, including pseudoaneurysms, presents challenges due to anatomical complexity and the risk of complications such as limb ischemia. The Western Trauma Association emphasizes the role of both open and endovascular techniques in these cases, with endovascular repairs particularly advantageous for pseudoaneurysms [27]. Endovascular approaches offer a minimally invasive option with favorable outcomes, as demonstrated in a study by Carbonell et al., which reported low complication rates and no cases of ischemia or amputation following endovascular treatment of axillary-subclavian arterial trauma [28].

Furthermore, research by Chen et al. underscores that surgical exploration and repair, whether by open or endovascular techniques, effectively relieve symptoms and restore normal radial artery pulse in patients with traumatic axillary artery pseudoaneurysms [25]. This highlights the importance of timely intervention to prevent further ischemic damage and improve functional recovery.

In our scenario, we chose open surgical repair, given that our patient is young, free of comorbidities, and in need of a durable vascular solution. This decision parallels the management of axillary artery pseudoaneurysms, where open repair allows for direct vessel reconstruction and the use of autologous grafts, ensuring long-term patency and reduced risk of reintervention. Nonetheless, follow-up for this patient was limited to one month, and no formal functional scoring was applied, which should be acknowledged when interpreting outcome durability.

## Conclusions

These two cases illustrate the value of individualized management strategies in vascular trauma, while also highlighting the limitations of generalizing from isolated experiences. The choice between endovascular and open surgical repair must consider patient-specific factors, including age, comorbidities, and the nature of injury. While our cases align with the broader literature suggesting that endovascular techniques offer a minimally invasive alternative with reduced perioperative risks, particularly beneficial for elderly patients with multiple comorbidities, this observation should be interpreted cautiously, given the small sample size and short follow-up. On the other hand, open surgery remains essential for cases requiring immediate and extensive vascular reconstruction, particularly in younger patients with fewer comorbidities. However, long-term outcomes could not be fully assessed in our report.

A major challenge in vascular trauma management is the absence of large-scale clinical trials and standardized guidelines, leading to variability in treatment approaches. Current recommendations are largely based on retrospective analyses and expert consensus, underscoring the need for further research to establish clear, evidence-based protocols. Until such data are available, a case-by-case approach remains essential.
